# Hybrid Filtering Compensation Algorithm for Suppressing Random Errors in MEMS Arrays

**DOI:** 10.3390/mi15050558

**Published:** 2024-04-24

**Authors:** Siyuan Liang, Tianyu Guo, Rongrong Chen, Xuguang Li

**Affiliations:** Key Laboratory of Information Communication Network and Security, School of Communications and Information Engineering, Xi’an University of Posts and Telecommunications, Xi’an 710121, China; telestorm@163.com (S.L.); taronion@163.com (R.C.); lxg_hk@163.com (X.L.)

**Keywords:** MEMS array, wavelet threshold denoising, BPNN, data fusion, FPGA online testing

## Abstract

To solve the high error phenomenon of microelectromechanical systems (MEMS) due to their poor signal-to-noise ratio, this paper proposes an online compensation algorithm wavelet threshold back-propagation neural network (WT-BPNN), based on a neural network and designed to effectively suppress the random error of MEMS arrays. The algorithm denoises MEMS and compensates for the error using a back propagation neural network (BPNN). To verify the feasibility of the proposed algorithm, we deployed it in a ZYNQ-based MEMS array hardware. The experimental results showed that the zero-bias instability, angular random wander, and angular velocity random wander of the gyroscope were improved by about 12 dB, 10 dB, and 7 dB, respectively, compared with the original device in static scenarios, and the dispersion of the output data was reduced by about 8 dB in various dynamic environments, which effectively verified the robustness and feasibility of the algorithm.

## 1. Introduction

MEMS have become an important part of different navigation systems due to their small size, low cost, low power consumption, and high impact resistance, and they have been widely used in attitude control devices, unmanned aerial vehicles, robot navigation, and satellite systems. However, the MEMS inertial navigation devices’ disadvantage of having significant errors restricts their use in high-end fields. As a result, it is common to analyze the measurement errors of MEMSs and increase their output accuracy as much as feasible, so that they can be employed in high-end fields. The errors of MEMS inertial devices can be divided into deterministic errors and random errors. Deterministic errors mainly refer to zero-point offset, scale factor, etc., which can be improved through calibration tests [1,2]. While random errors have uncertainty and randomness [3], which cannot be eliminated through calibration.

Random errors are a system noise-driven output that can be viewed as a non-smooth time series. Various methods have been proposed to reduce random errors, and the current methods for suppressing a single gyroscope fall into two main categories: conventional statistical methods, and artificial intelligence (AI) methods. The conventional statistical methods have been intensively studied by many scholars. There have been a large number of related studies and results, including wavelet denoising (WTD) [4], empirical modal decomposition (EMD) [5], Kalman filtering (KF) [6], etc. The WTD method not only has good time-frequency localization and multi-resolution characteristics, but also does not require error modeling. Therefore, it is widely used in medical [7] and mechanical systems [8] for non-smooth signal processing, as well as in marine technology [9]. However, the WTD method is essentially a Fourier transform with an adjustable window, so it still does not overcome the limitations of the Fourier transform, which is simple and less adaptable for handling at different scales. With the rapid development of artificial intelligence technology, it has been widely used in many fields, such as electric fluid power pumps [10] and human–computer interaction [11]. In particular, in MEMS error compensation technology, AI shows significant advantages. In order to establish a more accurate nonlinear model to cope with random errors, a variety of methods have appeared. For example, support vector machine [12,13,14] modeling technology, neural network [15,16] modeling technology, and so on.

The raw gyroscope signal is processed as a time series in both statistical and artificial intelligence (AI) methods, and a model is either defined or trained to compensate for noise-induced errors. The performance of statistical methods is limited by the fixed model parameters and traditional AI methods have limited learning capabilities. Therefore, hybrid approaches are one of the most intuitive solutions for enhancing denoising performance. In [17], wavelets and neural networks were combined for the offline processing of gyroscope signals. In [18], UKF was merged with RNN for real-time filtering of gyroscope random errors. In [19], the trend term of MEMS gyroscope data was first extracted using empirical modal decomposition (EMD), and then an adaptive Kalman filter (AKF) was used to compensate for the random errors of the MEMS gyroscope. These methods can model and compensate for the random errors as accurately as possible for a single gyroscope. However, the accumulated noise of MEMSs is large during long-term operation, and the current processing technology and fabrication process cannot reduce the system noise of MEMS gyroscopes well in a short time period [20], and it is difficult to improve the output accuracy of a single gyroscope, so forming a single gyroscope into a gyro array [21] and using a form of fusion filtering to improve its output accuracy has been the subject of research by academics both domestically and internationally in recent years.

MEMS arrays are digitally filtered and fused using multiple sensor data to obtain an optimal virtual MEMS output [22,23]. Chang et al. studied the signal fusion problem of gyroscope arrays based on simplified and typical models of gyroscopes [24,25,26] and designed a system consisting of six gyroscopes. Song et al. designed redundant 3-gyro, 5-gyro, and 8-gyro systems [27] and proposed an optimized filtering scheme for gyroscope array fusion to improve the fusion accuracy. Although it is becoming increasingly common to combine virtual gyro techniques with other filtering techniques, most studies are combined with conventional statistical methods, relatively few reports are combined with neural networks, and even fewer reports are based on hybrid methods to build more accurate models for real-time estimation and compensation of random errors in MEMS arrays.

In response to the above phenomenon, this paper adopts 10 MEMSs to form a complete array structure, in order to improve the output accuracy of a MEMS gyro array and system reliability for the purpose of research, for the random error of a single MEMS gyro to design a WT-BPNN hybrid filtering algorithm. At the same time, the support matrix method is used to fuse the array data. Finally, we successfully deployed the WT-BPNN hybrid filtering algorithm, as well as the support matrix fusion algorithm, in the MEMS array developed based on ZYNQ. In this process, the compensation of the random error of a single MEMS gyro became the top priority of the research. The WT-BPNN hybrid filtering algorithm is a hybrid filtering algorithm that directly combines wavelet threshold denoising and a BP neural network. First was the preprocessing process, where we utilized the wavelet threshold denoising technique to process the raw data, with the purpose of effectively separating the useful information and noise in the MEMS gyro data. Through this step, we successfully reduced the degree of discreteness of the original data, which provided a more stable and reliable data base for the subsequent training of the BPNN; second, the process of the BPNN compensating for the preprocessed data and the optimal network model were derived through multiple simulations and training. Thus, a fixed network model was obtained and encapsulated into a function written into the FPGA project. In order to verify the feasibility and validity of the model, online validation was performed. During the validation process, the RS422 serial protocol was used for communication, and the results showed that the model performed well in practical applications and achieved the expected results. The following are the main contributions of this paper:

1. Combination of wavelet and BPNN: The data output from an MEMS gyroscope has obvious random time-varying characteristics. Wavelet threshold denoising, as a fixed algorithm, is less flexible, while neural networks can analyze uncertain nonlinear systems more efficiently and improve the modeling accuracy of gyro drift through their own learning ability, without a priori information of the system model. Since BPNNs are more sensitive to data quality, this paper utilized wavelet threshold denoising to preprocess the data, which greatly improved the performance of the model.
2. BPNN improvements: First, based on the data characteristics of the MEMS array model, a suitable network structure was designed, using the minimum number of network layers, as well as the minimum number of neurons, to achieve the best compensation effect. Second, in order to reduce the problem of local minima of the BPNN, the additional momentum method was introduced to optimize it, because this has the effect of momentum, which helps the network to cross the local minima point in the parameter space.
3. Development of embedded systems: We wrapped the trained BPNN model into a function that was ported under the FPGA hardware project. The feasibility of the WT-BPNN hybrid filtering algorithm proposed in this paper was tested online in a variety of settings using the ZYNQ7010 model.

The remainder of this essay is organized as follows: The second part provides an overall description of the algorithmic system. The model of the neural network created through simulation is thoroughly explained in the third section. Part IV gives the online experimental results and the analysis based on field programmable gate array (FPGA). The fifth part concludes the paper.

## 2. Algorithm System Overview

**Figure 1:**
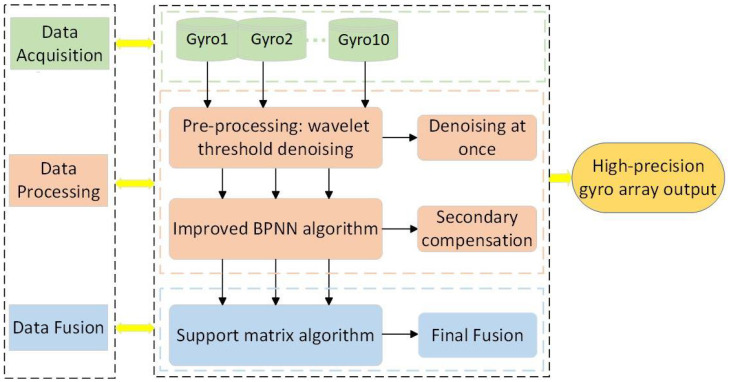
General flow of random error compensation algorithms for MEMS arrays. This includes three main parts: data acquisition, data processing, and data fusion.

The MEMS array random error compensation algorithm consists of three main parts: data acquisition, data processing, and data fusion, to achieve high-precision applications for low-precision gyros, as shown in Figure 1.

**Figure 2:**
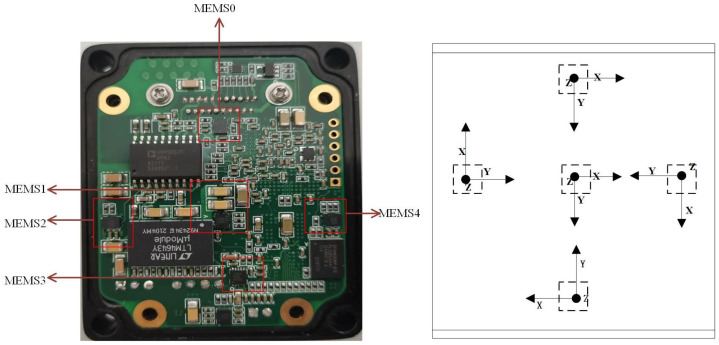
Gyroscope array PCB board. A front and back side arrangement was used (one side is shown in the figure).

A sensor array is a crucial technique for increasing the output precision of sensors. In order to increase output precision, 10 MEMSs were utilized in this study to construct a full array structure. The MEMS array arrangement was taken as a forward and reverse configuration, as illustrated in Figure 2. The array provided us with an original dataset for BPNN model learning based on the additional momentum method, and the validation of the MEMS array random error compensation algorithm was performed in many different scenarios.

The random errors of a single MEMS gyroscope are

(1) $$\begin{array}{c} \left\{\begin{array}{c} y(t)=\omega (t)+b(t)+n(t)\\ \dot{b}\left(t\right)=\omega_{b}\left(t\right) \end{array}\right. \end{array}$$

where *y* is the gyro output, ω is the input rate signal, *b* is the bias drift caused by rate random wander (RRW) $\omega_{b}$, and n is the white noise of angular random wander (ARW). Extending this model to a 10-group gyroscope array, the virtual gyro is modeled as:

(2) $$\begin{array}{c} \left\{\begin{array}{c} Z={\left[1,1,\cdots 1\right]}_{1\times N}^{T}\cdot \omega +b+V\\ \dot{b}=W_{b} \end{array}\right. \end{array}$$

where Z = $\left[\begin{array}{c} y_{1}\\ \vdots \\ y_{N} \end{array}\right]$, b = $\left[\begin{array}{c} b_{1}\\ \vdots \\ b_{N} \end{array}\right]$, $W_{b}\;=\left[\begin{array}{c} \omega_{b1}\\ \vdots \\ \omega_{bN} \end{array}\right]$, V = $\left[\begin{array}{c} n_{1}\\ \vdots \\ n_{N} \end{array}\right]$.

**Figure 3:**
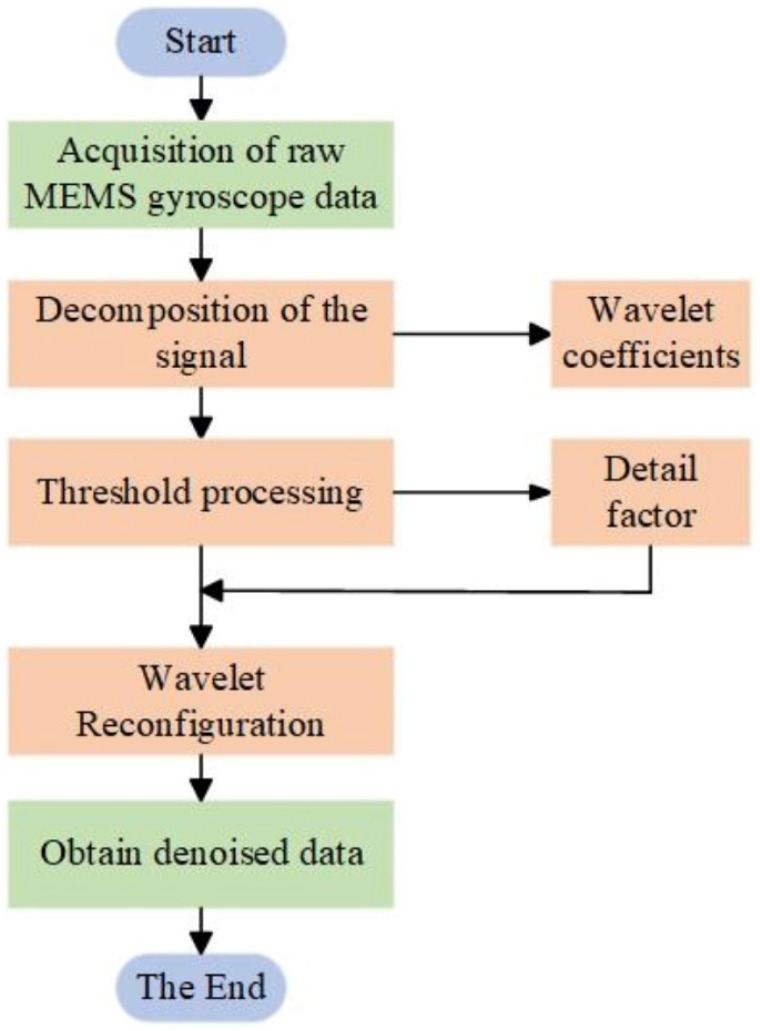
Wavelet denoising flow chart. The overall scheme of wavelet thresholding for the first round of denoising the original data.

In this study, we used wavelet threshold denoising to perform the first round of processing on the raw data output from the MEMS gyro array. Wavelet thresholding is a time-frequency domain transform that is capable of multi-scale transformation of the signal and noise reduction according to the distribution characteristics of the noise in the frequency domain, and divided into three parts to complete the operation, as shown in Figure 3:

1. Decompose the original MEMS signal using wavelet transform, select the appropriate wavelet basis, determine the number of wavelet decomposition layers N, process the signal, and obtain the wavelet coefficients;
2. Selecting a suitable threshold function and processing the detail components to obtain new detail coefficients;
3. The wavelet reconstruction of the signal is performed based on the low-frequency coefficients in layer N of the wavelet decomposition and the high-frequency coefficients in layers 1 to N, after quantization to obtain the denoised MEMS signal.

Because of the diversity of wavelet basis functions, using different wavelet bases to analyze the same problem will produce different results, so the selection of the optimal wavelet base is a very important issue in practical applications. The wavelet basis chosen for this topic was Daubechier (dbN).

Selecting the appropriate wavelet threshold is the key to wavelet denoising, and the commonly used threshold functions can be divided into soft thresholding and hard thresholding, both of which are easy to use but have their drawbacks. Hard thresholding typically involves setting wavelet coefficients smaller than the threshold value to 0, while wavelet coefficients larger than the threshold value are left at their original values. This causes the function to discontinue, causing the signal to oscillate and have a lower smoothness than the previous signal, which may contain new noise and appear pseudo-Gibbs-like. The soft threshold shrinkage transform has better continuity, but the processed signal will cause distortion, such as edge blurring. To address the above problems, targeted improvements were made in this study, and the new thresholding function is

(3) $$\omega_{m}=\left\{\begin{array}{c} \omega -\omega {\left|\frac{\lambda }{\omega }\right|}^{\frac{\left|\varphi \right|}{\lambda }},\left|\omega \right|\geq \lambda \\ 0,\left|\begin{array}{c} \omega \end{array}\right|<\lambda \end{array}\right.$$

The improved threshold function is between soft and hard thresholding, which avoids the abovementioned shortcomings and greatly optimized the pre-processed data. This data were then used to train a BP neural network model based on the additional momentum method.

A BP neural network is a multilayer feedforward network trained through error backpropagation (referred to as error backpropagation), and its algorithm is called the BPNN algorithm, where the basic idea is to search for the optimal value in data using the gradient descent method. The BPNN algorithm includes two processes of signal forward propagation and error backpropagation; that is, calculating the error output in the direction from input to output, and adjusting the weights and thresholds in the direction from the output to the input. In forward propagation, the input information is first mapped to the hidden layer, and then operated using nonlinear transformation to produce the output signal, and if the actual output does not match the desired output, it is transferred to the backward propagation process of the error. The error back-propagation is used to back-propagate the output error through the hidden layer to the input layer-by-layer, and to apportion the error to all units in each layer, using the error signal obtained from each layer as the basis for adjusting the weights of each unit. By adjusting the linkage strength of the input nodes to the hidden layer nodes and the linkage strength of the hidden layer nodes to the output nodes, the input and output signal error values are adjusted according to the linkage strength threshold, so that the error decreases along the gradient direction, and the network parameters (weights and thresholds) corresponding to the minimum error are determined after repeated training and then saved.

**Figure 4:**
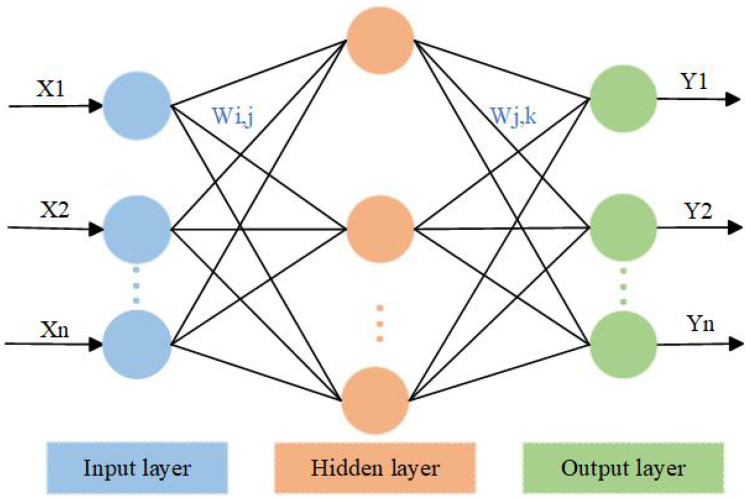
Network topology diagram. The basic structure of a neural network.

Figure 4 depicts the three-layer structure of the BPNN: the input layer, hidden layer, and output layer. Where the input layer is known data and the output layer is used to output model predictions. The hidden layer can contain a single layer or multiple layers, and the most basic BPNN usually has only one hidden layer.

BPNNs are widely used in function approximation, pattern recognition, classification, and data compression.

A BPNN has the advantage of adaptive learning and can construct mathematical models after several autonomous learning and testing phases. The computational load of the BPNN is fully compatible with the high-rate processing of a gyro array output.

A BPNN has the abovementioned benefits, but because of the internal effect of the BPNN, it also has the drawbacks of sluggish convergence, limited convergence accuracy, and a propensity to fall into local minima when the network is operating. Owing to the problems of BPNNs, we introduced the method of additional momentum, so that it can cross these local minima to a great extent. This method allows the network to consider, not only the role of the error in the gradient, but also the influence of the changing trend on the error surface when revising its weights, and this improvement scheme is more advantageous in hardware applications than other algorithm improvements such as the introduction of the fruit fly algorithm, while the method is also more memory efficient. In this study, a value proportional to the previous weight change is added to each weight change, and a new weight change is generated according to the backpropagation method. The formula for weight adjustment with an additional momentum factor is

(4) $$\begin{array}{c} ▵W_{ij}\left(k+1\right)=\left(1-mc\right)\eta \delta_{i}P_{j}+mc▵W_{ij}\left(k\right) \end{array}$$

(5) $$\begin{array}{c} ▵b_{i}\left(k+1\right)=\left(1-mc\right)\eta \delta_{i}+mc▵b_{i}\left(k\right) \end{array}$$

where *k* is the number of trainings; mc is the momentum factor, which took the value of 0.96 in this study.

**Figure 5:**
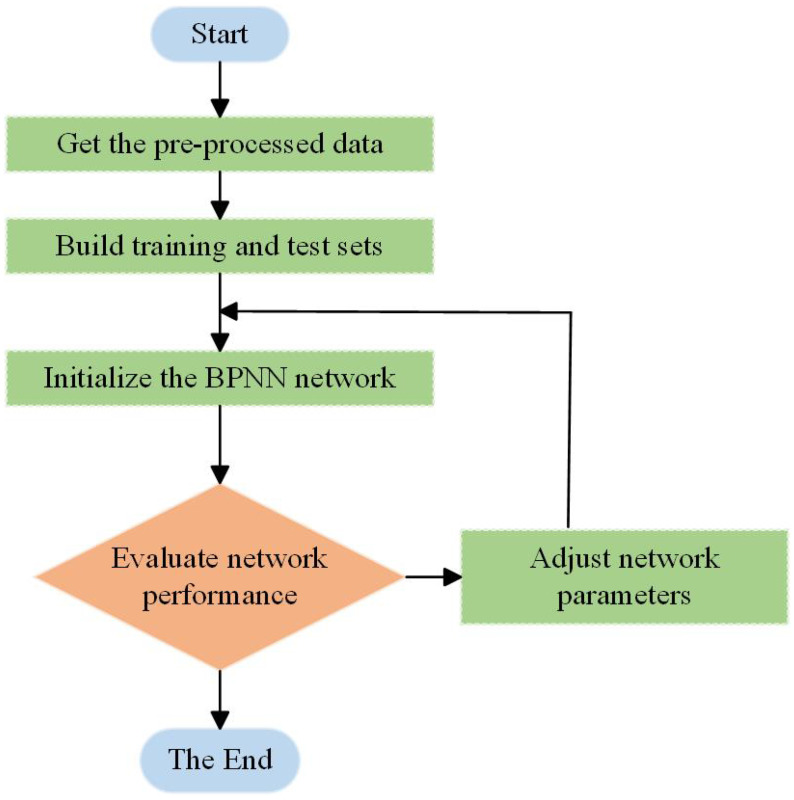
Neural network algorithm processing flow. The data after wavelet threshold denoising were used for error compensation with this flowchart.

This BPNN model that introduces an additional momentum method, not only improves the training efficiency compared to the traditional BPNN, but also jumps out of the local minima to find a better solution.The primary denoised data were modeled using a BPNN model based on the additional momentum method, and Figure 5 depicts the process of training and adjusting the network’s parameters, which was a crucial step in this paper’s algorithm. The Allan variance was used to evaluate the network output data, and the evaluation took into account not only the Allan variance output curve but also the running time. The most suitable network for an engineering application was finally selected and deployed on a MEMS array hardware device.

We filtered most of the noise through wavelet threshold denoising and using a BPNN model based on the additional momentum method for an individual MEMS with random errors. For the processing of the remaining small portion of noise, we used the support matrix fusion method to improve the output accuracy.

The mutual support degree matrix between sensors is as follows:

(6) $$\begin{array}{c} D=\left[\begin{array}{ccc} d_{11} & \cdots & d_{1j}\\ \vdots & \ddots & \vdots \\ d_{i1} & \cdots & d_{ij} \end{array}\right] \end{array}$$

The combined support function is

(7) $$\begin{array}{c} S_{i}=\sum_{j=1,j\neq i}^{n}r_{ij},i=1,2,\ldots n \end{array}$$

where $S_{i}$ denotes the reliable decision of other gyroscopes on the *i*th gyroscope. Consistency measure function:

(8) $$\begin{array}{c} \omega_{i}\left(k\right)=\frac{N_{i}}{n-1},i=1,2,\ldots ,n,0<\omega_{i}\left(k\right)\leq 1 \end{array}$$

## 3. Random Error Compensation Experiment

### 3.1. Simulation Data Processing

To verify the performance of the algorithm proposed in this paper, a simulation validation environment was conducted. First, the effect of wavelet thresholding on the first round of data denoising is discussed. We chose the original data from the X-axis and discuss the number of decomposition layers using the wavelet base of db3. From Table 1, we can observe that as the number of decomposition layers increased, the variance of the gyroscope data became smaller and better. Although the variance was further reduced, some useful information might have been lost, resulting in signal distortion, and the processing time became longer and longer as the number of decomposition layers increased. We considered the real-time processing of a large amount of data in the actual project for the processing speed requirements. Consequently, we decided on 5 as the number of breakdown layers.

The data after one round of denoising were compensated using the BPNN model based on the additional momentum method, and we discuss the effect of single-layer as well as multi-layer approaches. As shown in Table 2, the data appeared overfitted when the number of layers was too high, and we chose a three-layer network by considering the compensation effect as well as engineering applications.

### 3.2. Simulation Result Measurement

The angular random wander noise, zero bias instability noise, and rate random walk are important parameters to measure the performance of gyroscopes. Allan variance is a classical time-domain analysis technique that is widely used to evaluate the performance of gyroscopes, and this analysis method can clearly show the performance change before and after noise reduction.The data variance can reflect the degree of dispersion of the data and evaluate the noise suppression effect of the algorithm. Therefore, in this chapter as well as in the online real-world test section in Section 4, we use Allan’s variance as well as the data variance to evaluate the WT-BPNN hybrid filtering algorithm proposed in this paper.

**Figure 6:**
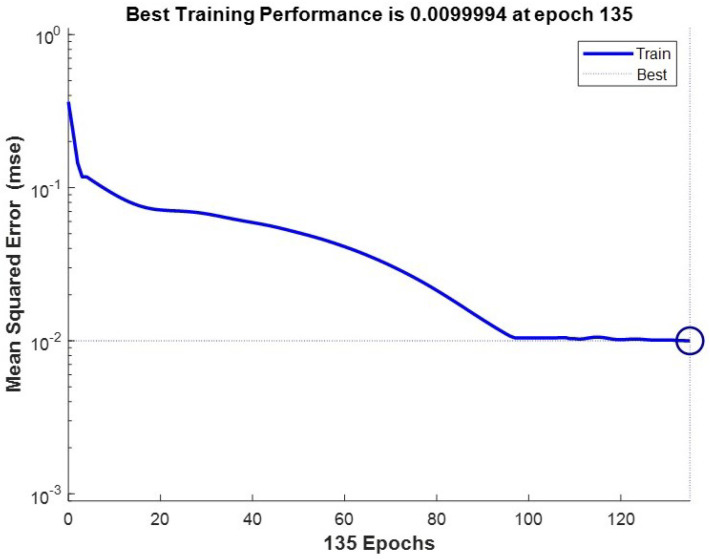
BPNN training loss.

The development board was preheated at room temperature and the X-axis gyroscope output data were collected at rest. A large amount of data were collected for training, and the Allan variance of each result was compared to determine the most suitable network training model, setting the learning rate of this study to 0.01, the number of hidden layer nodes to 15, the minimum error of learning target to 0.0003, and the number of iterations to 5000; Figure 6 shows the loss curve under this model.

**Figure 7:**
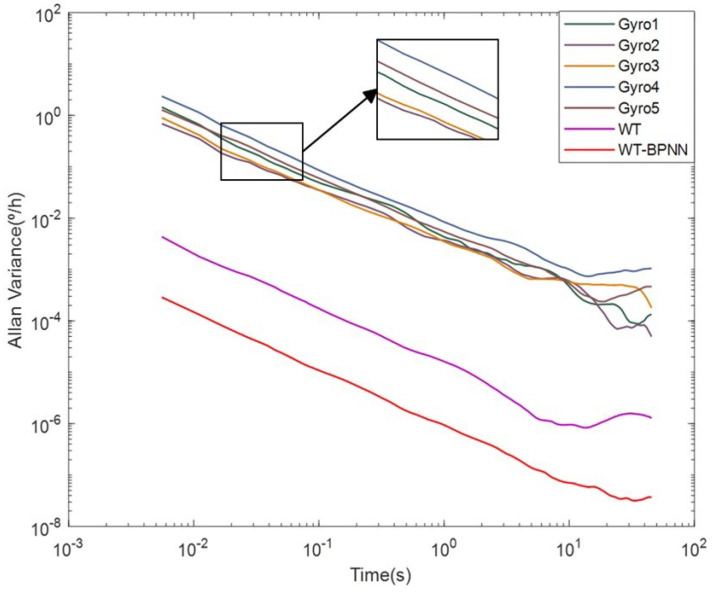
Comparison of Allan analysis.This figure depicts the simulation of the WT-BPNN hybrid filtering algorithm on the x-axis.

In order to visualize the difference between the processed data and the original data, five gyroscope data points were selected from the sensor array for visualization. The results are shown in Figure 7. The result of the WT-BPNN hybrid filtering algorithm was the corresponding curve of the “virtual gyroscope”. Compared with the original data, the curve trend was basically the same, indicating that the data processing did not change the error characteristics of the MEMS array. The error magnitude was greatly reduced compared with the original data, which indicated that the various errors of the gyroscope were significantly reduced, which was sufficient to prove the effectiveness of the algorithm.

The noise figure obtained from Allan variance processing is shown in Table 3. From Table 3, it can be seen that the WT-BPNN hybrid filtering algorithm improved about 14.12 dB, 11.71 dB, and 8.03 dB in angular random wander N, zero bias instability B, and angular rate random wander K, respectively.

Through a comprehensive analysis of Figure 7 and Table 3, we can see that the angular random wandering coefficient and zero bias instability factor were small, which indicates that the stability of the detection mode was good, and the various error coefficients in the Allan variance were significantly reduced, which indicates that the method could effectively reduce the random error of the MEMS gyro performance and enhance the accuracy of the MEMS gyro output data, as well as the WT-BPNN hybrid filtering algorithm processing. The effect was significantly better than for the traditional algorithm.

Table 4 shows the results of the comparison between the variance in the original data and the optimization results. From the table, it can be seen that the performance of the WT-BPNN hybrid filtering algorithm was significantly better than the traditional algorithm, and the variance in the WT-BPNN hybrid filtering algorithm was reduced by 15.64 db compared with the minimum value of the original data, and the convergence was also significantly improved.

**Figure 8:**
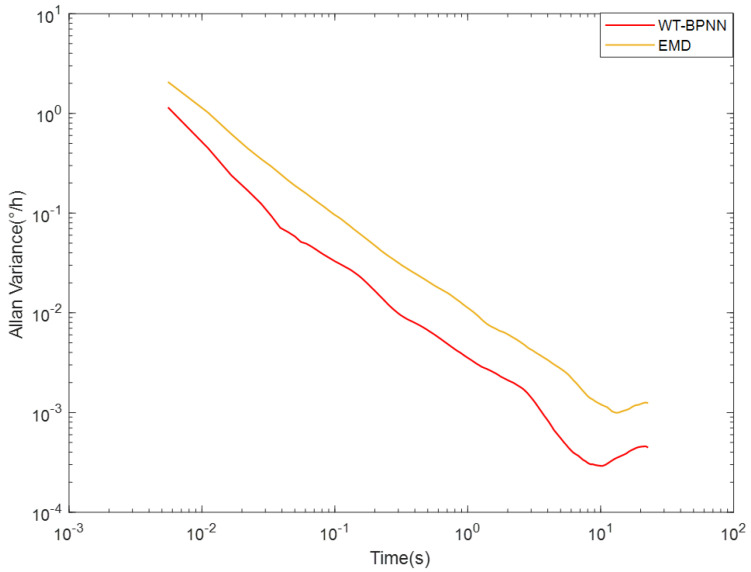
Comparison plot of Allan variance for different algorithms.

To further verify the superiority of the algorithm, we compared it with the EMD algorithm. The Allan variance curve is shown in Figure 8 and the data variance results are shown in Table 5.

From Figure 8, the overall curve of WT-BPNN hybrid filtering algorithm decreased compared with EMD algorithm, indicating that this paper’s algorithm was significantly better than EMD algorithm. From Table 5, the variance in the data processed by the WT-BPNN hybrid filtering algorithm was smaller than that processed by the EMD algorithm, indicating that the algorithm in this paper had a better effect in reducing the dispersion of the original data.

## 4. Fpga-Based Online Experiments

### 4.1. Test Environment Introduction

**Figure 9:**
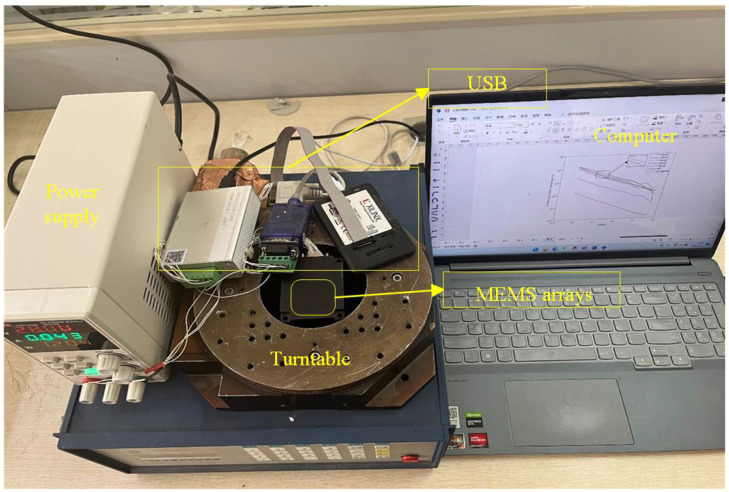
Experimental environment. Online testing of all hardware and software devices.

In order to verify the feasibility and generalization ability of the above model, we encapsulated the trained BPNN model into a function, converted it into C to add it into a project file, and deployed it on a MEMS array development board for ZYNQ-based online compensation testing. The experimental setup is shown in Figure 9 and consisted of a MEMS array test platform, which included a six-axis IMU sensor LSM6DS0 from ST, a turntable, a power supply, and a computer system with Xilinx SDK 2019.1 installed, where the LSM6DS0 used a gyroscope with a practical range of ±500 dps.

All experiments were tested in the same external environment, and the data collected during the experiments were acquired at room temperature. The MEMS was fixed on the turntable and the power supply output was 28 V and 0.043 A when the MEMS array was connected. The control software was run on a computer to monitor the data acquisition process and acquire and store the data before and after the algorithm processing. The sampling frequency was set to 200 HZ.

We gathered the unprocessed X, Y, and Z axes raw data, wavelet processed data, and WT-BPNN hybrid filtering algorithm processed data, in that order.

### 4.2. Actual Measurement Results

#### 4.2.1. Static Experiments

To verify the denoising effect of the proposed algorithm in static experiments, the MEMS array system was preheated for 30 min, and then the data were collected, including the X/Y/Z axis MEMS gyro array output data collected at room temperature. The acquired data were imported into Matlab for analysis after the outliers were removed. We selected five sets of raw data (including maximum and minimum values) for each axis for visual comparison and analysis.

**Figure 10:**
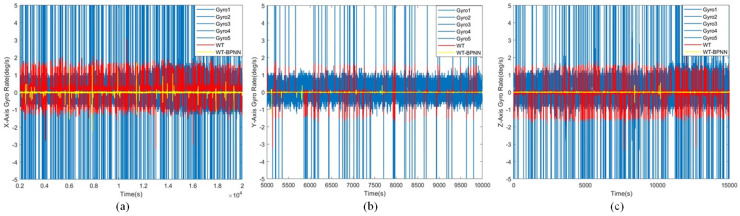
This figure shows a comparison of the original signal, the soft and hard combined wavelet threshold denoised signal, and the denoised data of our proposed algorithm. (**a**) shows the data of the X-axis, (**b**) shows the data of the Y-axis, and (**c**) shows the data of the Z-axis.

Figure 10 shows a comparison of the original signal with the denoised signal. It is evident from these figures that the WT-BPNN hybrid filtering method significantly reduced the noise. We quantitatively analyzed the noise reduction effect of the model using Allan variance to clearly demonstrate its efficacy.

**Figure 11:**
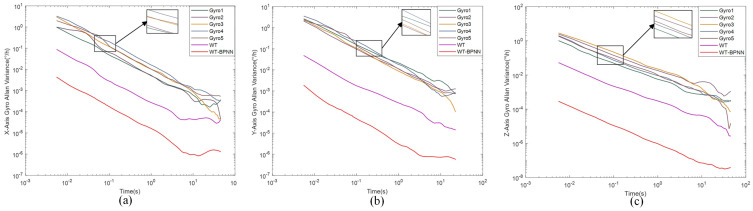
This figure shows the Allan analysis curve, which contains the original signal, the soft and hard combined wavelet threshold denoising signal, and the denoising data of our proposed algorithm. (**a**) shows the data of the X-axis, (**b**) shows the data of the Y-axis, and (**c**) shows the data of the Z-axis.

Figure 11 shows the Allan variance comparison results, and Table 6 shows comparison plots of the denoised signal and the original signal parameters.

For the X-axis gyro, it can be seen from Figure 11a and Table 6 that the angular random wander N, zero bias instability B, and angular rate random wander K were improved by about 12.33 dB, 10.25 dB, and 6.57 dB, respectively; for the Y-axis gyro, it can be seen from Figure 11b and Table 6 that the angular random wander N, zero bias instability B, and angular rate random wander K were improved by about 9.49 dB, 8.26 dB, 6.17 dB; for Z-axis gyro, it can be seen from Figure 11c and Table 6 that the improvement in angular random wander N, zero bias instability B, and angular rate random wander K was about 9.43 dB, 8.50 dB, and 7.12 dB, respectively.

The various error coefficients in the Allan variance analysis were significantly reduced, indicating that the random errors could be effectively limited after processing the data with the WT-BPNN hybrid filtering algorithm proposed in this paper. Through the analysis of the X/Y/Z-axis data, we found that the model had better performance for the X-axis in the hardware test and was closer to the effect obtained from the simulation test, while it also had an obvious effect for the Y/Z-axis, but was less effective than the X-axis. We speculated that this was because the X-axis data were used exclusively in the training of the network to make it have a better compensation ability for X-axis; however, the model had a good generalization ability for the static data of gyro arrays and a high compensation ability for static errors, as seen in the comprehensive analysis of experiments.

Table 7 shows a comparison of the results of the variance in X/Y/Z three-axis data. From the data in the table, it can be concluded that the WT-BPNN hybrid filtering algorithm could effectively reduce the degree of dispersion of the original data, indicating the effectiveness of the algorithm.

#### 4.2.2. Dynamic Experiments

The working environment for dynamic verification was carried out under constant temperature and pressure experimental conditions. We used a laboratory single-axis turntable to rotate at a fixed angular rate and collected gyro-constant velocity data at 1°/s, 3°/s, and 5°/s, and analyzed the stability by comparing the standard deviation of the original data, after wavelet threshold denoising, and after processing by the WT-BPNN hybrid filtering algorithm. The effectiveness of the WT-BPNN algorithm designed based on the static gyro data was verified, and the details are shown in Table 8 below.

The above table shows that the algorithm was the generalizable for X/Y/Z axes, and its stability improved by 13.48 dB, 10.14 dB, and 10.24 dB, respectively, in the stationary state. The encapsulated algorithm was verified from multiple angles at low speed, and the data stability was increased by around 8 dB, demonstrating the effectiveness of the encapsulated algorithm in terms of dynamics, as well as showing that its expressive power was smooth and outperformed the neural network to a certain degree. The above experiments show that we could make the hybrid filtering model have a good compensation effect in many different scenarios through one PC learning training.

We successfully verified the static effect of the model on FPGA hardware devices, as well as the dynamic effect of the low-speed constant part, and the experimental results showed the effectiveness of the algorithm.

Since our hybrid filtering model needs to be applied in engineering, the algorithm design takes into account, not only the compensation effect on random errors, but also the operation cost and the operation time. The fusion filtering algorithm was tested in a laboratory environment, and the overall design of the algorithm was found to be feasible. The device operated smoothly for stationary and various low-speed scenarios. When the speed was increased or the MEMS array was in irregular motion at high speed, the standard deviation of the data analysis still met our desired value, but the mean value was biased towards the range of the data at rest, thus deviating from the true value we wanted. This shows that the algorithm was unable to accurately capture and compensate the raw data during high-speed motion, which is an issue we need to study in depth subsequently.

## 5. Conclusions

In this paper, we conducted an in-depth study on the problem of improving the output accuracy of random errors of MEMS arrays, designed a WT-BPNN hybrid filtering algorithm, and deployed it in MEMS arrays developed based on ZYNQ, in which gyroscopes were taken as the main object of study. We realized the real-time and high-precision output of the gyro array in multiple scenarios on the hardware platform by learning and training from raw data. In terms of technical contribution, we proposed the WT-BPNN hybrid filtering algorithm, which preprocesses the data by wavelet thresholding and then compensates the data by utilizing a BPNN model based on the additional momentum method. Unlike with the research methods of most other scholars, in addition to combining the traditional algorithm with neural networks, we also applied it to MEMS arrays to further improve its output accuracy. We evaluated the proposed method on an original collected dataset, as well as on the MEMS array development board in our lab. During the laboratory tests, there was no phenomena such as data latency and good stability, and the output accuracy of the gyroscope was substantially improved when analyzing the measured data, which demonstrated the overall suitability of the algorithm of the project. Thus, this algorithm enables long-term stability and reliability of tracking and localization systems for outdoor multi-sensor devices.

However, this paper had the following limitations, the BPNN model based on the additional momentum method was encapsulated into a function written in the FPGA project, the training parameters were fixed, and due to the limitation of the laboratory equipment, we did not perform an in-depth study of the complex dynamic data, which caused some limitations in capturing all the dynamic data. Therefore, the method performed poorly in high-speed motion and irregular motion. In the future, we hope to conduct comprehensive training on static as well as various dynamic data, to obtain a more universal hybrid filter, optimize the model so that data changes in motion states can be captured quickly, and extend it to accelerometer applications.

## Figures and Tables

**Table 1:** Wavelet decomposition layer experiment.

Number of Wavelet Decomposition Layers	3	5	6	9
Standard deviation	0.5526	0.3109	0.2968	0.2895
Processing time/S	0.15	0.16	0.23	0.24

**Table 2:** Comparison of single-layer and multi-layer BPNNs.

Number of Network Layers	1	3	5
Standard deviation	0.0117	0.0036	0.0097
Processing time/S	1.1	1.9	5.1

**Table 3:** Raw data and optimization results.

Noise Types		Angle Random Walk °/$h^{2/1}$	Zero-Bias Instability °/h	Rate Random Walk °/$h^{2/3}$
Original signal	MAX	0.0820	0.1610	0.0752
	MIN	0.0340	0.0963	0.0146
WT		0.0096	0.0119	0.0115
WT-BPNN		0.0013	0.0065	0.0023
Improved		14.12 dB	11.71 dB	8.03 dB

**Table 4:** Comparison of variance between the original data and optimized results.

Algorithm Analysis	Original Signal		WT	WT-BPNN
	MAX	MIN		
Data variance	0.1054	0.0881	0.0096	0.0024

**Table 5:** Comparison of variance of different algorithms.

Algorithm Analysis	EMD	WT-BPNN
data variance	0.0395	0.0157

**Table 6:** X/Y/Z-axis raw data and optimization results.

	Noise Types		Angle Random Walk °/$h^{2/1}$	Zero-Bias Instability °/h	Rate Random Walk °/$h^{2/3}$
X-axis gyro	Original signal	MAX	0.0859	0.0847	0.0152
		MIN	0.0308	0.0159	0.0019
	WT		0.0237	0.0139	0.0060
	WT-BPNN		0.0018	0.0015	0.000418
Y-axis gyro	Original signal	MAX	0.1060	0.0531	0.0345
		MIN	0.0169	0.0087	0.0014
	WT		0.0133	0.0083	0.0036
	WT-BPNN		0.0019	0.0013	0.000338
	Improved		9.49 dB	8.26 dB	6.17 dB
Z-axis gyro	Original signal	MAX	0.1473	0.1532	0.0925
		MIN	0.0149	0.0078	0.0016
	WT		0.0050	0.0043	0.0041
	WT-BPNN		0.0017	0.0011	0.000310
	Improved		9.43 dB	8.50 dB	7.12 dB

**Table 7:** Comparison of the variance in the data in the X/Y/Z axes.

Algorithm Analysis	Original Signal		WT	WT-BPNN
	MAX	MIN		
X-axis gyro	0.2054	0.0269	0.0032	0.0016
Y-axis gyro	0.1796	0.0924	0.0056	0.0020
Z-axis gyro	0.2597	0.0856	0.0102	0.0031

**Table 8:** Dynamic data standard deviation analysis.

		Original Signal				WT	WT-BPNN	Proportion
	Parameters	MAX		MIN				
		Average	Standard Deviation	Average	Standard Deviation	Standard Deviation	Standard Deviation	
X-axis gyro	Stationary	0.6676	1.8864	0.1452	0.0831	0.3197	0.0027	14.88dB
	1 rad/s	1.0120	0.2080	0.9939	0.1500	0.0407	0.0222	8.30 dB
	3 rad/s	3.1652	0.2597	2.9766	0.1159	0.0394	0.0152	8.82 dB
	5 rad/s	5.1014	0.2097	4.9728	0.0961	0.0337	0.0159	7.81 dB
	Stationary	0.5889	1.1556	0.2731	0.1583	0.1944	0.0071	13.48 dB
Y-axis gyro	Stationary	0.5138	1.5329	0.1037	0.2625	0.2160	0.0254	10.14 dB
	1 rad/s	1.1699	0.2477	1.0346	0.1560	0.0860	0.0206	8.79 dB
	3 rad/s	3.2623	0.6428	2.8677	0.3084	0.0945	0.0312	9.95 dB
	5 rad/s	5.0394	0.2126	4.8536	0.1462	0.0704	0.0212	8.39 dB
	Stationary	0.7331	1.0230	0.2481	0.2043	0.2579	0.0163	10.98 dB
Z-axis gyro	Stationary	0.7497	1.3545	0.2494	0.0349	0.2222	0.0033	10.24 dB
	1 rad/s	1.2158	0.3254	1.0188	0.1894	0.0582	0.0237	9.03 dB
	3 rad/s	3.1045	0.5114	2.8543	0.2788	0.0632	0.0429	8.12 dB
	5 rad/s	5.0536	0.2495	4.8769	0.1835	0.0892	0.0366	7.00 dB
	Stationary	0.6627	1.1327	0.2455	0.1771	0.2635	0.0095	12.70 dB

## Data Availability

The original contributions presented in the study are included in the article, further inquiries can be directed to the corresponding authors.
